# Palmitic Acid Targets Human Testicular Peritubular Cells and Causes a Pro-Inflammatory Response

**DOI:** 10.3390/jcm9082655

**Published:** 2020-08-17

**Authors:** Artur Mayerhofer, Kim-Gwendolyn Dietrich, Henryk F. Urbanski, Frank-Michael Köhn, Ulrich Pickl, Matthias Trottmann, Paul Kievit, Harald Welter

**Affiliations:** 1 Biomedical Center, Cell Biology, Anatomy III, Ludwig Maximilian University Munich, D-82152 Planegg-Martinsried, Großhaderner Straße 9, 82152 Munich, Germany; kim.dietrich@bmc.med.lmu.de (K.-G.D.); welter@bmc.med.lmu.de (H.W.); 2 Division of Neuroscience, Oregon National Primate Research Center, Beaverton, OR 97006, USA; urbanski@ohsu.edu; 3 Andrologicum München, 80331 Munich, Germany; info@andrologicum.com; 4 Urologie und Andrologie am Promenadeplatz, 80333 Munich, Germany; u.pickl@web.de (U.P.); praxis@urologe-androloge.de (M.T.); 5 Division of Cardiometabolic Health, Oregon National Primate Research Center, Beaverton, OR 97006, USA; kievitp@ohsu.edu

**Keywords:** high-fat diet, human male fertility, inflammation, obesity, palmitic acid, testis

## Abstract

Palmitic acid (PA) is a major fatty acid, derived from diet and endogenous production, which is being linked to inflammation. While such actions of PA at the level of the testis remain difficult to examine, we reasoned that studies in human testicular cells may be instructive. Human testicular peritubular cells (HTPCs) can be isolated from men and cultured. They have contractile properties but also produce Interleukin 6 (IL6), express the inflammasome member NLRP3, and via glia cell line derived neurotrophic factor (GDNF), they contribute to the spermatogonial stem cell niche. We found that PA at 100 µM significantly increased the levels of *IL6,* while *NLRP3* or the related Interleukin 1 beta (*IL1beta*) were not affected. The contractility marker calponin (*CNN1*) and the growth factor *GDNF* were likewise not affected. ELISA studies confirmed the stimulatory PA actions on IL6. Hence, PA derived from diet and/or endogenous sources may be able to foster a pro-inflammatory milieu in the testis. A possible link of these results to diet and high fat intake and obesity is indicated by the about 12-fold elevated testicular levels of *IL6* in testes of obese rhesus monkeys (*n* = 3), fed with a Western Style diet. They had elevated 2–5-fold increased body fat and increased circulating triglyceride levels. Further consequences of PA and obesity for testicular functions remain to be evaluated.

## 1. Introduction

There is evidence for a sterile type of inflammation in the testes of men suffering from idiopathic infertility [1,2,3]. The changes are typical for a pro-inflammatory tissue environment and are predominantly observed in the peritubular wall. This small testicular compartment in healthy, fertile men consists of elongated smooth muscle-like peritubular cells, i.e., contractile cells, which transport sperm and secrete extracellular matrix [3,4]. In contrast, in infertile men, fibrotic remodeling of the wall and immune cells, i.e., tryptase- and, in part, chymase-positive mast cells and CD68-positive macrophages, which also express tumor necrosis factor alpha (TNFalpha), are among the obvious changes. In addition, phenotypic changes of peritubular cells, namely a loss of smooth muscle characteristics occur [5,6,7,8,9,10]. The data suggest that the peritubular wall and its cells play roles in male infertility.

Peritubular cells are not well examined cells. However, they can be isolated from patients (HTPCs) and were previously characterized (e.g., [2,11,12,13]). It became clear that they are more than contractile cells. They produce e.g., glial cell lined derived factor (GDNF), required for spermatogonial stem cell (SSC) renewal [14], but also a number of pro-inflammatory factors, including interleukin 6 (IL6), and other cytokines, and they express the inflammasome family member NLRP3 (see [2,12,13,15]) and Toll-like receptors (TLRs; [1]). Thus, peritubular cells contribute to diverse testicular functions, including immunological properties of the testis and may fuel events that contribute to male sub/infertility.

Fatty acids (FAs) stem from ingested lipids and are produced by de novo-synthesis. Saturated FAs, particularly stearic, lauric, and palmitic acid (PA; C16:1), the latter being the main product of activity of fatty acid synthase, are known to activate TLR2 and 4. This activation was shown to drive pro-inflammatory responses and expression of pro-inflammatory genes (see [16,17,18,19]). Whether lipids and FAs/PA may target testicular peritubular cells and hence, whether they may be involved in the induction of testicular inflammation in man, is not known. In rodents, PA acts at the level of the male gonad, namely at Sertoli cells [20], where induction of dysfunction and apoptosis were reported [21,22].

Increased adiposity and high-fat diet (HFD) are linked to chronic systemic inflammation [23,24]. Increased circulating levels of pro-inflammatory cytokines, e.g., TNFalpha, Interleukin 1beta (IL1beta), and IL6 are reported [25,26]. In the context of the male gonad, IL6 is of interest, as it is involved in the regulation of the blood-testis barrier, formed by Sertoli cells. Elevated levels may lead to its breakdown, as shown in a rodent study [27]. The consequences may include exposure of spermatogenic cells to blood-borne harmful agents and induce autoimmune events [28], with deleterious consequences on sperm production.

While the mentioned data mainly stem from animal studies, there is evidence for adverse effects of obesity on reproduction in men [29,30]. They include lower testosterone levels and lower sperm counts, (see [31]). The mechanisms are under discussion and neuroinflammation causing a dysregulation of the hypothalamus–pituitary–gonadal axis, specifically at the level of the GnRH neurons (see [31]) was suggested as one possible reason.

Other possible actions, e.g., ones exerted by PA at the level of the human testis, are not well known and remain difficult to examine in man. We reasoned that studies employing HTPCs may be instructive and therefore we examined actions of PA in cultured HTPCs. In addition, expression levels of inflammatory genes in testes of three obese monkeys with elevated body fat and high circulating triglycerides were evaluated.

## 2. Materials and Methods

### 2.1. Human Samples: HTPCs

As described, the HTPCs studied were isolated from small testicular fragments [12]. The study was approved by the local ethical committee (Ethikkommission, Technische Universität München, Fakultät für Medizin, project number 309/14 and project 491/18S-KK). All patients provided written informed consent. The experiments were carried out in accordance with the relevant guidelines and regulations. Derived cells were cultured in DMEM High Glucose (Gibco, Paisley, UK) with 10% fetal bovine serum (Capricorn Scientific, Ebsdorfergrund, Germany), 1% penicillin/streptomycin (Biochrom, Berlin, Germany) at 37 °C and 5% (*v*/*v*) CO_2_. The cells studied in frame of this study stem from a total of 14 individual donors, with diagnoses ranging from obstructive azoospermia and normal spermatogenesis to non-obstructive idiopathic azoospermia. The age of the donors ranged from 29 to 55 years. All cells were initially characterized, and we verified that they express typical HTPCs markers, including androgen receptor (*AR*) and smooth musle actin (*ACTA2*).

### 2.2. Samples from Normal and Obese Monkeys

Rhesus monkey testis mRNA was obtained from unrelated studies approved by the Oregon National Primate Research Center (ONPRC) Institutional Animal Care and Use Committee. Obese animals were maintained on a high-fat diet (HFD) consisting of 36% fat (mostly saturated fats from lard; TAD Primate Diet—5LOP, Test Diet, St. Louis, MO, USA) that is supplemented with calorically dense treats (5LOP pellets, peanut butter, honey, banana, and cornstarch). Control animals were maintained on a diet consisting of 13% fat (from soybean oil, Monkey Diet 5045, Lab Diet, St. Louis, MO, USA).

Testicular RNA used for RT-PCR was extracted from 6 lean adult monkeys (age range 20–24 years, body weight range 9–11 kg) and 3 obese (age range 13–18 years, body weight range 16–24 kg).

### 2.3. Stimulation Experiments with Palmitic Acid (PA)

The stimulation with PA (Sigma Aldrich (P9767), St. Louis, MO, USA) was conducted as follows: 30.6 mg PA were dissolved in 150 mM NaCl and 0.17 mM BSA (Ultra Fatty Acid Free BSA, Roche 10775835001) to reach a 1 mM PA working solution (final ratio of 6:1). A solution of 150 mM NaCl/BSA served as solvent control. After performing pilot studies (with cells from 4 donors) for the experiments described here, HTPCs were incubated with/without 100 µM PA for 24 h in serum free medium. In all experiments a solvent control was included, which allowed us to compare the results of the treatment. The PA concentration of 100 µM was chosen in pilot experiments because it did not negatively affect viability of HTPCs and because the reported plasma concentration of FAs in a HFD can reach 100–400 µM or more (see [32]). Experiments (treatment and control) were done with cells from individual donors and then repeated with cells from other donors. For the qPCR studies cells from 6 donors were used, for the ELISA studies cells from additional 4 donors were studied.

### 2.4. Isolation of RNA, Reverse Transcription (RT-PCR) and Quantitative Real Time PCR (qPCR)

All procedures used were described previously [12]. In brief, RNeasy Plus Micro Kit (Qiagen, Hilden Germany) was used for total RNA isolation from HTPCs in passages 7–10 [12] and 200 ng RNA were reverse transcribed, using random 15mer primer and SuperScript^TM^ II (Invitrogen, Darmstadt, Germany). A LightCycler^®^ 96 System (Roche) and QuantiFast^®^ SYBR Green PCR Kit (Qiagen) were used. Primer information is given in Table 1. Unless indicated otherwise, primers matched human and rhesus monkey sequences. Non-reverse transcription control and non-template control were used as negative controls.

### 2.5. IL6 ELISA Measurements

The assay (Platinum Elisa Human IL6, #BMS213, affimetrix eBioscience, Bender MedSystems GmbH, Vienna, Austria) was performed, as described earlier (see [1,33]). Cellular supernatant of control and PA-treated cells (24 h) were derived from 4 donors. Samples were run in duplicates and values were normalized to cellular protein.

### 2.6. Statistical Analysis

The results of qPCRs were analyzed according to the 2^−ΔΔCq^ method as described earlier (Walenta et al., 2018) and expression levels were normalized to *RPL19*, which served as endogenous reference.

Results were normalized to the controls (control treatment of HTPCs or non-obese samples). They are depicted as means ± SEM. Statistical analysis was done by *t*-tests (two-tailed) of ΔΔC_q_ values and ELISA results, using GraphPad Prism 6.0 Software (GraphPad Software, San Diego, CA, USA).

## 3. Results

### 3.1. Effect of PA on HTPC mRNA Level of IL6, NLRP3, IL1beta and CNN1

**Figure 1:**
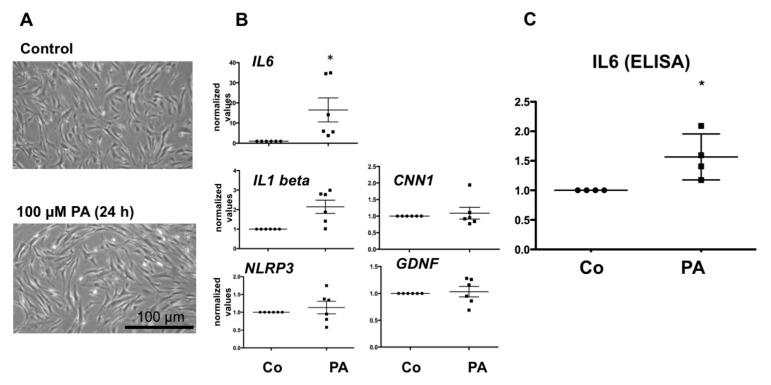
Effect of a 24 h incubation period with 100 µM palmitic acid (PA) on human testicular peritubular cells (HTPCs) compared to the corresponding solvent control (Co) on cellular morphology (**A**), two representative pictures are shown: (**B**) mRNA level of Interleukin 6 (*IL6*), Interleukin 1 beta (*IL 1beta*), the inflammasome *NLRP3,* calponinin (*CNN1*) and glial cell lined derived factor *(GDNF*), (**C**) IL6 secretion of HTPCs. *n* = 6 for mRNA and *n* = 4 for IL6 protein; individual values of the treatment group are shown, as well as the mean ± SEM; values were normalized to the respective solvent control. Asterisk denotes statistical significance, * *p* < 0.05.

As shown in Figure 1A, the cellular morphology of HTPCs cultured with 100 µM PA for 24 h did not change compared to cells cultured in the presence of the solvent control. Under both conditions, cells were characterized by a slim, spindle-like shape. Using qPCR, we found that PA at 100 µM significantly (*p* < 0.05) increased mRNA levels of *IL6*, while *NLRP3* and *IL1beta* were not significantly affected (*p* > 0.05) (Figure 1B). The levels of the contractility marker *CNN1* and the growth factor *GDNF* were likewise not changed.

### 3.2. Effect of PA on IL6 Secretion in HTPCs

ELISA measurements confirmed the stimulatory PA actions of IL6 in 4 samples (Figure 1C), thus supporting the qPCR results.

### 3.3. Obesity and Testicular IL6 Levels in Rhesus Monkeys

**Figure 2:**
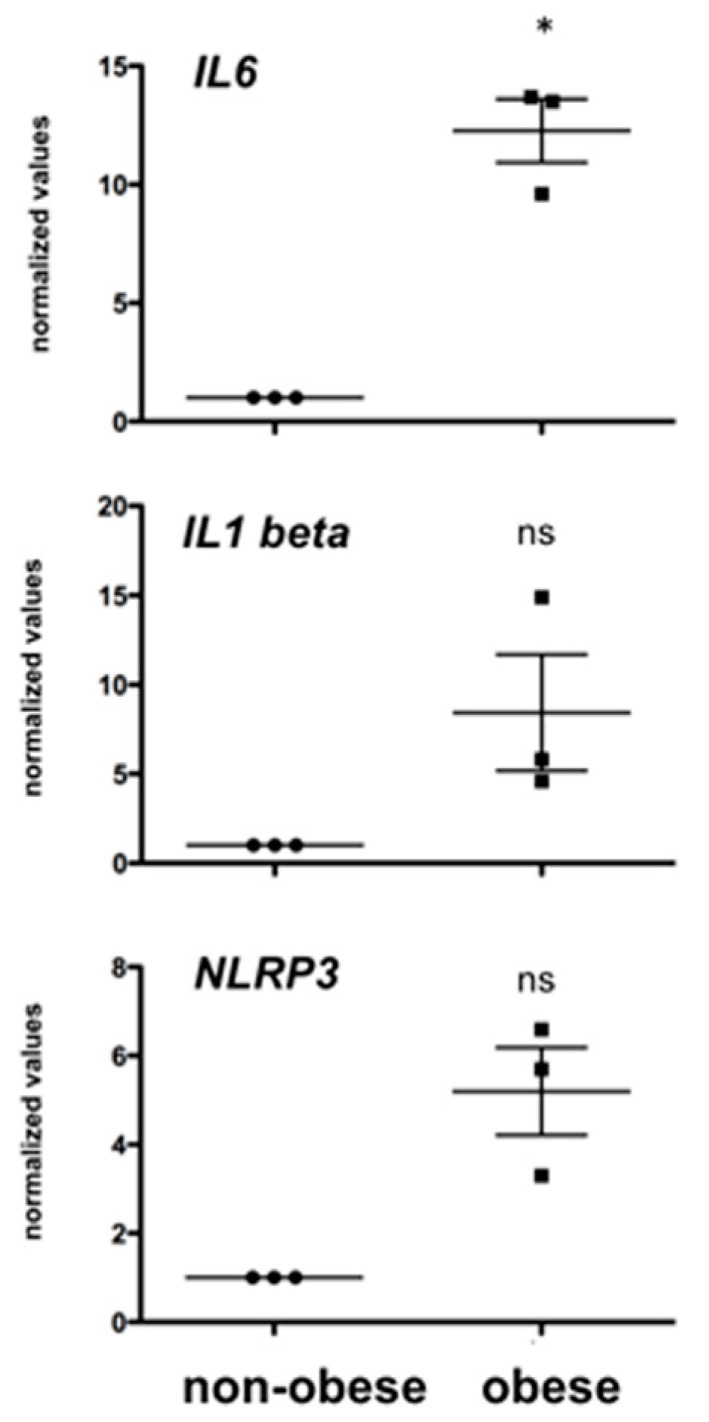
Levels of testicular Interkeulin 6 (*IL6*) were significantly elevated in testes of obese monkeys, while higher levels of Interleukin 1 beta (*IL1beta*) or the inflammasome *NLRP3* did not reach statistical significance; *n* = 3, * *p* < 0.05; ns: not statistically significant. The data of the obese animals (*n* = 3) are given as individual levels and as the mean ± SEM. The values were normalized to the levels of the controls group (pooled results of *n* = 6 non-obese monkeys).

To explore consequences of obesity on testicular inflammatory markers, qPCR studies were performed using samples from obese monkeys and monkeys with normal body weight as controls. Levels of *IL6* in the testes of the three obese adult Rhesus monkeys fed with a Western Style diet were almost 12-fold elevated (*p* < 0.05) compared to one in an age-matched control group (Figure 2). Levels of *IL1beta* and *NLRP3* were also elevated but did not reach significance levels (*p* > 0.05).

## 4. Discussion

There is ample evidence for adverse effects of obesity on reproduction in men, namely lower testosterone levels and lower sperm counts [29,30,31,34]. A meta-analysis study in a large cohort of men, for example, came to the conclusion that obesity is positively correlated with a higher prevalence of azoospermia or oligozoospermia [35]. Another study reports that a high BMI in men is positively correlated with reduced semen parameters [36].

The underlying reasons for obesity-related impaired male reproductive function, specifically impaired spermatogenesis, are likely multifaceted and the chains of events remain difficult to examine in men, bearing in mind the complex regulation of testicular functions on one hand. On the other hand, obesity is also correlated with a host of diseases, including metabolic disease [16]. Medical conditions and medication, next to diet, may therefore also have a say and may result in impaired testis functions [37,38,39,40,41].

Rodent models are often used in biomedical research, including obesity research. Yet, results of a recent gene expression study of several organs clearly revealed that the testis, together with the liver, shows the largest differences in the expression of individual genes of all organs examined between the mouse and human species [42]. This emphasizes that in order to understand the human situation, adequate models for the human testis are required. Furthermore, the striking differences between the single-layered peritubular wall in rodents and the multilayered peritubular compartment in men and monkeys indicate that studies on peritubular cells require an adequate model.

In our study we therefore turned to an established cellular model, HTPCs, which are the only cells that can be isolated from the human testes, cultured and then examined in vitro. We employed them to explore the consequences of 100 µM PA. We tested possible actions on a smooth muscle marker, on *GDNF*, and inflammatory factors. PA is indeed well known to cause inflammation (e.g., [17,18,19]) and we found that PA elevates *IL6* transcript and IL6 protein levels in HTPCs. Heterogeneity in basal and secreted IL6 levels of the human cells was observed, which is in line with the results of previous studies in HTPCs, in which IL6 was examined [1,6]. This may be due to the difference in age of the patients, differences in life style, and/or underlying reason for sub-/infertility. In contrast, PA did not significantly affect other HTPC-parameters under the experimental condition, including *GDNF* or the smooth muscle marker *CNN1*.

An HFD is linked to high circulating levels of FFA [32] and the reported plasma concentration of FFAs can reach up to 100–400 µM or even more. The concentration used in our study is in this range. Hence, provided that the cellular response is also present in the body, HTPCs may contribute to a pro-inflammatory milieu in the testis.

Evidence for such a milieu to exist in situ, in testes samples from three obese monkeys, with elevated blood triglyceride levels, was obtained, albeit in a limited set of samples. We found strikingly high expression levels of *IL6* in the testes of obese monkeys. While elevated testicular *IL6* in (whole) testes samples may stem from many testicular sources, peritubular cells are based on or result in HTPCs, likely included. Of note, other inflammatory parameters (*NLRP3*), also expressed by Sertoli cells; [2] and *IL1beta* showed a clear tendency to higher values, but due to the low number of available samples, they did not reach significance level.

Obesity in man is not only associated with impaired male reproductive function, but is also well known to be associated with low-grade, chronic inflammation, as reflected by alterations of immune cell populations and tissue remodeling in organs, such as liver, pancreas, and blood vessels [43]. Evidence for a sterile type of inflammation in the human testis was reported in cases of male idiopathic infertility due to impaired spermatogenesis [1,13]. The present results obtained in HTPCs and in a translational nonhuman primate model, suggest that obesity and/or increased dietary intake of PA, via induction of inflammation, could lead to a pro-inflammatory environment and possibly to impairments of testicular functions. Additional studies are now required to examine these points and elucidate the mechanisms and further consequences.

## Figures and Tables

**Table 1:** Nucleotide sequence of primers (Gene and gene name) employed in real time PCR including amplicon size and reference ID.

Gene	Gene Name	Reference ID	Nucleotide Sequence	Amplicon Size
*mk* *IL6*	Interleukin 6	NM_001042733.2	5′-TGGCTGAAAAAGATGGATGCT-3′ 5′-TTGCTGCTCACTACTCTCAAACCT-3′	134
*mk* *IL1 β*	Interleukin 1 beta	NM_001042756.1	5′-AAAGCTTGGTGATGTCTGGTC-3′ 5′-GGACATGGAGAACACCACTTG-3′	89
*NLRP3*	NLR family, pyrin domain containing 3	NM_001114351.1	5′-GTGTTTCGAATCCCACTGTG-3′ 5′-TCTGCTTCTCACGTACTTTCTG-3′	143
*IL6*	Interleukin 6	NM_000600.4	5′-AACCTGAACCTTCCAAAGATGG-3′ 5′-TCTGGCTTGTTCCTCACTACT-3′	159
*CNN1*	Calponin	NM_001308342.2	5′-CGAAGACGAAAGGAAACAAGGT-3′ 5′-GCTTGGGGTCGTAGAGGTG-3′	186
*GDNF*	Glial cell line derived neurotrophic factor	NM_000514.3	5’-GCAGACCCATCGCCTTTGAT-3′ 5′-ATCCACACCTTTTAGCGGAATG-3′	93
*RPL19*	Ribosomal protein L19	NM_000981	5′-AGGCACATGGGCATAGGTAA-3′ 5′-CCATGAGAATCCGCTTGTTT-3′	199
